# Complete Genome Sequence of an Alphacoronavirus from Common Vampire Bats in Peru

**DOI:** 10.1128/MRA.00742-20

**Published:** 2020-08-20

**Authors:** Laura M. Bergner, Richard J. Orton, Daniel G. Streicker

**Affiliations:** a Institute of Biodiversity, Animal Health, and Comparative Medicine, College of Medical, Veterinary, and Life Sciences, University of Glasgow, Glasgow, United Kingdom; b MRC-University of Glasgow Centre for Virus Research, Glasgow, United Kingdom; DOE Joint Genome Institute

## Abstract

Bats host diverse coronaviruses, including taxa capable of pandemic spread in humans. We report the genome of an alphacoronavirus from a neotropical bat species (*Desmodus rotundus*) in Peru, which contributes to our understanding of bat coronaviruses in nature.

## ANNOUNCEMENT

Coronaviruses (CoVs) (family *Coronaviridae*) are positive-sense, single-stranded RNA viruses that naturally circulate in many vertebrates. CoVs are relatively common and genetically diverse in bats and include zoonotic species that cause severe acute respiratory syndrome (SARS-CoV), Middle East respiratory syndrome (MERS-CoV), and CoV disease 2019 (SARS-CoV-2) (1–5). Due to the geographic origin of the most prominent zoonotic CoVs, most knowledge of bat CoVs is derived from Old World species. Nonetheless, comparatively limited sampling has revealed diverse CoVs among bats in North and South America (6–9). Additional knowledge of the diversity and distribution of CoVs in these species will aid efforts to understand virus ecology within bat reservoirs, anticipate zoonotic risk, and accelerate identification of reservoir hosts following emergence.

We report the genome of an alphacoronavirus from common vampire bats (Desmodus rotundus, family *Phyllostomidae*), termed DesRot/Peru/Amazonas/CoV (isolate AMA_L_F). The genome was derived from shotgun sequencing of 10 pooled samples collected with noninvasive rectal swabs from D. rotundus bats from two colonies in Rio Escondido, Amazonas, Peru (ENA accession number ERR2756788) (10). Briefly, total nucleic acid was extracted using a BioSprint One-for-All veterinary kit (Qiagen) and a Kingfisher 96 Flex system. Following DNase I (Ambion) treatment, rRNA depletion (Ribo-Zero; Illumina), and cDNA synthesis (Maxima H Minus first-strand cDNA synthesis kit; Thermo Fisher Scientific), sequencing libraries were prepared using the KAPA DNA library preparation kit for Illumina (Kapa Biosystems). Sequencing was performed on an Illumina NextSeq 500 system (read length, 150 bp). A total of 17,760,709 raw reads were processed through an in-house pipeline, including quality filtering with Trim Galore v.0.4.0 and prinseq-lite v.0.20.4 (11, 12), assembly with SPAdes v.3.10.1 (13), and classification with DIAMOND blastx v.0.8.20 (14), leading to a genome of 29,097 bp with a mean coverage of 226.7 reads. BLASTn analysis against GenBank showed that the most similar full genome was that of an alphacoronavirus from a microbat (GenBank accession number MK472070.1) (70.7% nucleotide similarity); that virus was used to determine genome termini. The two genomes were of similar initial size (MK472070.1, 28,009 bp; DesRot/Peru/Amazonas/CoV, 29,140 bp), and the new genome aligned over the length of the reference with relatively few gaps (final untrimmed alignment, 29,887 bp). DesRot/Peru/Amazonas/CoV had a GC content (42.9%) and genomic organization of major open reading frames (ORFs) (5′-ORF1a/ORF1ab-S-ORF3-E-M-N-3′) similar to those of other alphacoronaviruses.

**FIG 1:**
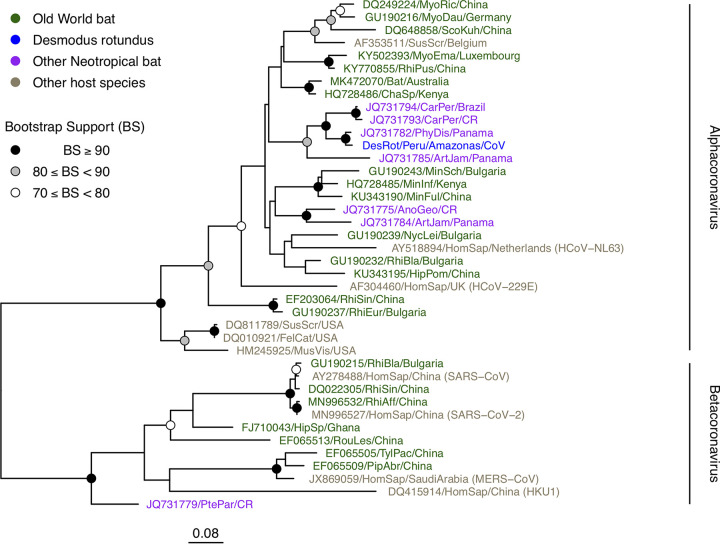
RdRp phylogeny including the novel vampire bat alphacoronavirus. The maximum likelihood tree was based on a 272-amino-acid alignment of 40 RdRp sequences, including the novel vampire bat CoV. Human-infecting species are indicated by names in parentheses. The scale bar represents the mean expected rate of substitutions per site. Node support is from 1,000 bootstrap replicates.

A 272-amino-acid section of the RdRp gene (5) was aligned with other representative CoVs using MAFFT v.7.017 (15). Maximum likelihood phylogenetic analysis performed in RAxML v.8.2.8 (16), using the LG+I+G substitution model identified by ProtTest 3 (17), showed that the vampire bat sequence was more closely related to other neotropical bat CoVs (96.2% nucleotide similarity, over 816 bp, to the most similar sequence [GenBank accession number JQ731782]) and fell within a clade of alphacoronaviruses from other *Phyllostomidae* bats (Fig. 1). Two Brazilian vampire bat sequences, which were within ORF1b but did not overlap completely with the RdRp section used for phylogenetic analysis, were compared to DesRot/Peru/Amazonas/CoV separately, displaying pairwise nucleotide identities of 69.2% over 52 bp (EU236685.1) and 98.1% over 572 bp (KU552072.1). In summary, DesRot/Peru/Amazonas/CoV is a genomic representative of neotropical bat alphacoronaviruses, providing a new resource for understanding the global diversity of CoVs.

### Data availability.

The complete genome sequence for DesRot/Peru/Amazonas/CoV has been deposited in GenBank under accession number MT663548. Raw data were deposited in the ENA under run accession number ERR2756788, experiment accession number ERX2769781, and study accession number PRJEB28138.
